# Advancement in utilization of magnetic catalysts for production of sustainable biofuels

**DOI:** 10.3389/fchem.2022.1106426

**Published:** 2023-01-10

**Authors:** Yutao Zhang, Weihua Li, Jialu Wang, Jiaxing Jin, Yixi Zhang, Jingsong Cheng, Qiuyun Zhang

**Affiliations:** ^1^ Engineering Technology Center of Control and Remediation of Soil Contamination of Guizhou Science and Technology Department, Anshun University, Anshun, Guizhou, China; ^2^ School of Chemistry and Chemical Engineering, Anshun University, Anshun, Guizhou, China; ^3^ College Rural Revitalization Research Center of Guizhou, Anshun University, Anshun, Guizhou, China

**Keywords:** magnetic, heterogeneous catalysis, reusability, esterification, transesterification, biodiesel

## Abstract

In this study, we summarize recent advances in the synthesis of magnetic catalysts utilized for biodiesel production, particularly focusing on the physicochemical properties, activity, and reusability of magnetic mixed metal oxides, supported magnetic catalysts, ionic acid-functionalized magnetic catalysts, heteropolyacid-based magnetic catalysts, and metal–organic framework-based magnetic catalysts. The prevailing reaction conditions in the production of biodiesel are also discussed. Lastly, the current limitations and challenges for future research needs in the magnetic catalyst field are presented.

## 1 Introduction

**FIGURE 1:**
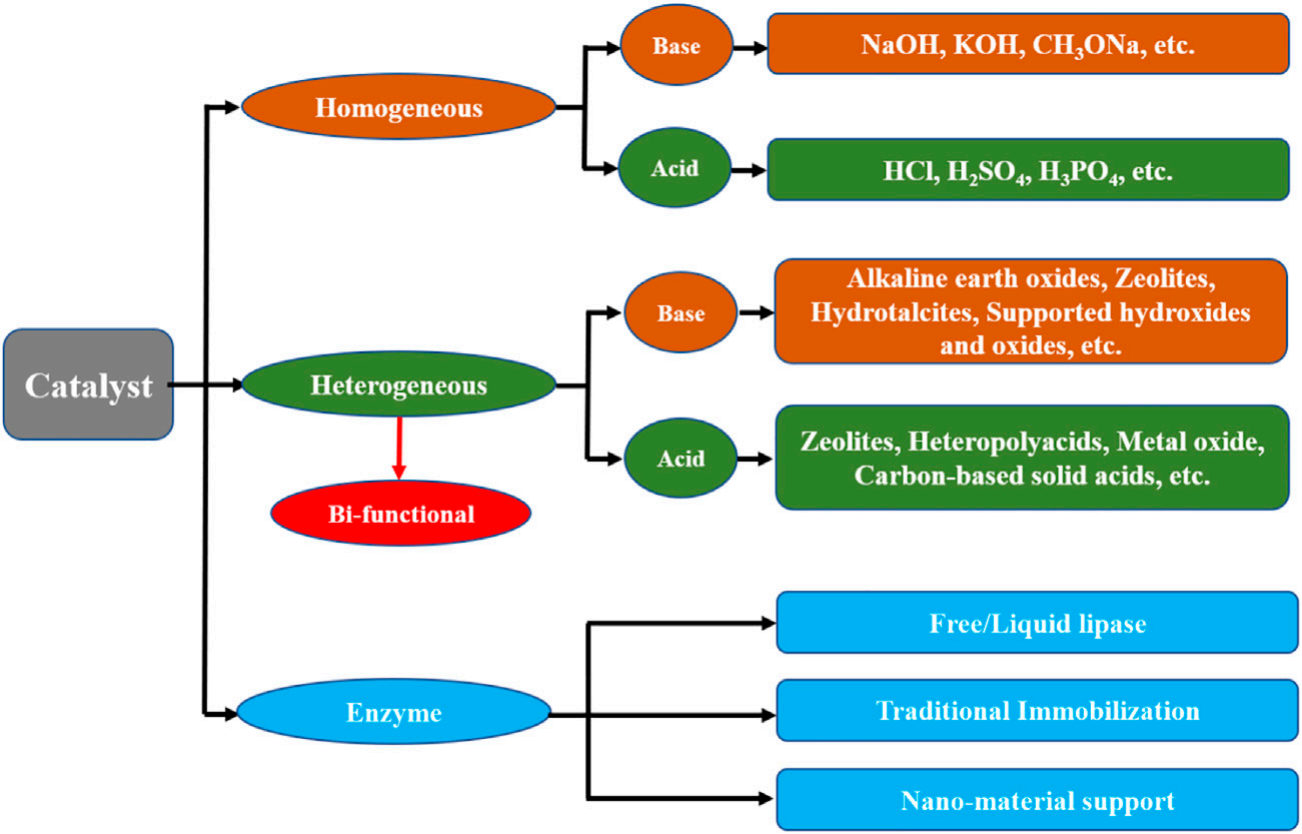
Classification of catalysts for biodiesel production.

With the rapidly expanding economy and high energy demand, the over-consumption of fossil fuels and fossil fuel usage has led to severe effects on the environment (e.g., global warming), creating wide attention among researchers (Li et al., 2023; Pan et al., 2022a; Zhang et al., 2022a; Pan et al., 2022b). Thus, seeking a sustainable energy resource is a high priority. To date, various types of biofuels, such as biodiesel, bioethanol, and aviation biofuels, have been considered as fossil fuel replacements. Among them, biodiesel (fatty acid alkyl ester, FAME) has been getting significant interest as an alternative fuel because of its safety, biodegradability, and carbon-neutrality (Zhang et al., 2020; Hoang et al., 2021). Currently, biodiesel is synthesized from free fatty acids (FFAs) and various oils mixed with short-chain alcohols, using homogeneous, heterogeneous, or enzymatic catalysts to promote the reaction (Figure 1) (Zhang et al., 2023). However, the homogeneous catalysis system exhibits numerous disadvantages, such as the fact that homogeneous catalysts (e.g., NaOH, KOH, H_2_SO_4_, etc.) are non-recyclable and cause pollution (Zhang et al., 2021; Liu et al., 2022). In contrast, heterogeneous catalysts (e.g., zeolites, heteropolyacids, metal oxides, etc.) have attracted growing interest owing to their low pollution and easy recovery (Woo et al., 2021; Zhang et al., 2022b; Paiva et al., 2022; Ul Islam et al., 2022). However, high-efficiency separation of the catalyst from the liquid phase and reduction of catalyst loss remain challenges. The use of magnetic separation techniques is an interesting approach to solving these problems (Chen et al., 2022).

In recent times, magnetic solid acid/base catalysts have been widely applied for esterification and transesterification reactions as compared to other heterogeneous catalysts because they are environmentally friendly and cheap, and their highly magnetic nature allows efficient separation with an external magnetic field (Shylesh et al., 2010; Zhang et al., 2014). The present work reviews recent applications of different types of magnetic catalysts and their functionalized magnetic composites employed in biodiesel production, including magnetic mixed metal oxides, supported magnetic catalysts, ionic acid-functionalized magnetic catalysts, heteropolyacid-based magnetic catalysts, and MOF-based magnetic catalysts, among others. The physicochemical properties, activity, and reusability of these magnetic catalysts are evaluated and discussed. Lastly, a brief conclusion and summary on the outlook for designing magnetic catalysts with high catalytic activity is presented.

## 2 Magnetic catalysts

In general, Fe-, Co-, and Ni-based catalysts exhibit permanent magnetism and can be used as magnetic materials; Fe-based catalysts have been especially widely studied. According to their characteristics, magnetic catalysts can be roughly classified into five types, namely, magnetic mixed metal oxides, supported magnetic catalysts, ionic acid-functionalized magnetic catalysts, heteropolyacid-based magnetic catalysts, and MOF-based magnetic catalysts.

### 2.1 Magnetic mixed metal oxides

Recently, spinel ferrites, MFe_2_O_4_ (where *M* indicates a transition metal atom of Cu, Zn, Mo, Co, or Mn) have been widely researched for applications as heterogeneous catalysts due to their thermal stability and ease of separation by using an external magnet. Luadthong et al. (2016) investigated the transesterification of palm oil using a copper ferrite spinel oxide (CuFe_2_O_4_) catalyst. The characterization results revealed that the major active species of CuFe_2_O_4_ were the Cu^2+^ and Fe^2+^. Optimal reaction conditions of 220°C, 1 g of catalyst, a methanol:oil molar ratio of 1:18, and a high FAME content of >90% were determined. A similar study was conducted by Ali et al. (2020), in which a cuprospinel CuFe_2_O_4_ catalyst was used for the transesterification of waste frying oil with methanol at 60°C, giving a 90.24% yield. Kinetic results showed that the transesterification reaction followed pseudo-first-order kinetics, and the activation energy was found to be 37.64 kJ/mol. AlKahlaway et al. (2021) prepared ferric molybdate, Fe_2_(MoO_4_)_3_, nanoparticles for biodiesel synthesis and the catalytic conversion of oleic acid was 90.5%.

In addition, some magnetic mixed metal oxides including MoO_3_/SrFe_2_O_4_ (Gonçalves et al., 2021), MnFe_2_O_4_/GO (Bai et al., 2021), MgFe_2_O_4_@OA@CRL (Iuliano et al., 2020), NaFeTiO_4_/Fe_2_O_3_–FeTiO_3_ (Gutierrez-Lopez et al., 2021), Mg^2+^-doped ZnFe_2_O_4_ (Ashok et al., 2021), and waste chalk/CoFe_2_O_4_/K_2_CO_3_ (Foroutan et al., 2022) have been explored for their application largely due to their unique magnetism. Gonçalves et al. (2021) prepared a magnetic catalyst, MoO_3_/SrFe_2_O_4_, for the transesterification of waste cooking oil, and results confirmed the success of MoO_3_ anchorage of the SrFe_2_O_4_ material. The activity test showed that a biodiesel yield of 95.4% was obtained in 4 h at 164°C. The MoO_3_/SrFe_2_O_4_ catalyst could be easily separated by a permanent magnet and showed high stability with a yield of 84% after five cycles. Bai et al. (2021) investigated the catalytic performance of a MnFe_2_O_4_/graphene oxide catalyst for biodiesel production from waste edible oil. The MnFe_2_O_4_/graphene oxide catalyst had a high basicity of 3978.6 mmol/g, and in transesterification reactions, a high biodiesel yield of 96.47% was achieved. Moreover, the physical properties of the synthetic biodiesel were within the ASTM D6751 and EN 14214 standard range. A K_2_CO_3_ modification to the waste chalk/CoFe_2_O_4_ was developed by Foroutan et al. (2022), and the characterization results showed that the composite catalyst had a lower surface area due to the introduction of K_2_CO_3_. The highest biodiesel yield of 98.87% was obtained under optimized conditions, and the activation energy and frequency factor of the reaction system was found to be 11.8 kJ/mol and 0.78 min^−1^, respectively.

Rezania et al. (2021) synthesized a heterogeneous magnetic MGO@MMO nanocatalyst via the ultra-sonication procedure for biodiesel production from waste frying oil. From the results, a high biodiesel yield of 94% was achieved with a 1.5 h reaction at 60°C; the catalyst could be separated and recycled four times, achieving an 86% biodiesel yield. However, after the eighth cycle, the biodiesel yield decreased significantly, possibly due to leaching of the active components or active site blocking. In a more recent study by Hanif et al. (2022), a magnetic Fe/SnO nanocatalyst supported on feldspar was synthesized for the transesterification of various non-edible oils. The magnetic catalyst exhibited a high catalytic activity with more than 97% yield for all the tested non-edible oils. A highly active bifunctional Na–Fe–Ca nanocatalyst was developed by Wang et al. (2022). The catalytic activity of the magnetic Na–Fe–Ca nanocatalyst in biodiesel production was evaluated at low temperatures. Interestingly, with a 500°C calcination temperature, the catalyst reached a 95.84% biodiesel yield with 16 cycles via magnetic separation. In conclusion, magnetic mixed metal oxides have been used successfully as acid/base catalysts or supports in the catalysis industry, and the design and composition of cheap, magnetic composite nanocatalysts is a highly desirable goal in the future.

### 2.2 Supported magnetic catalysts

Apart from magnetic spinel ferrite catalysts, supported magnetic acid/base catalysts have also attracted significant interest for biofuels production in recent years. At present, Fe_3_O_4_ magnetic particles do not commonly exhibit good catalytic activity, although they are easily separated and reused. Magnetic Fe_3_O_4_ is often used as a carrier, and the catalytic system is cost-effective and environment-friendly. Joorasty et al. (2021) prepared NaOH/clinoptilolite–Fe_3_O_4_ for the transesterification reaction of *Amygdalus scoparia* oil, and the highest biodiesel yield by the catalyst was 91%. The kinetics of NaOH/clinoptilolite–Fe_3_O_4_-catalyzed transesterification were also explored, and the activation energy was determined to be 9.21 kJ/mol. Xia et al. (2022) prepared bifunctional Co-doped Fe_2_O_3_–CaO nanocatalysts (Co/Fe_2_O_3_–CaO) and studied their catalytic performance in soybean oil transesterification. It was reported that the Co/Fe_2_O_3_–CaO catalyst had good ferromagnetism (26.2 emu/g) after the Co doping, and could be efficiently separated. In another study by Nizam et al. (2022), magnetic Fe_2_O_3_ immobilized on microporous molecular sieves (Fe_2_O_3_/MS) was developed using a plant extract-mediated method. In the catalytic reaction, the Fe_2_O_3_/MS catalyst exhibited excellent applicability in the esterification, transesterification, and photodegradation reactions. Mohamed et al. (2020) and Mohamed and El-Faramawy. (2021) used a newly developed *α*-Fe_2_O_3_/AlOOH(γ-Al_2_O_3_) nanocatalyst to produce biodiesel from waste oil. The *α*-Fe_2_O_3_/AlOOH(γ-Al_2_O_3_) catalyst presented the highest FAME yield and recyclability due to its large surface area of 323.3 m^2^/g, high acidity of 0.45 mmol/g, and exposed active site planes. Furthermore, thermal analyses showed that the catalytic reaction system was endothermic.

In a study conducted by Changmai et al. (2021a), a recoverable Fe_3_O_4_@SiO_2_–SO_3_H core@shell magnetic catalyst was successfully prepared by a stepwise co-precipitation, coating, and functionalization method. The obtained magnetic Fe_3_O_4_@SiO_2_-SO_3_H had a magnetic saturation of 30.94 emu/g, a relatively large surface area of 32.88 m^2^/g, and a high acidity of 0.76 mmol/g. The Fe_3_O_4_@SiO_2_–SO_3_H catalyst achieved a high conversion of *Jatropha curcas* oil of 98 ± 1% under optimal reaction conditions. Mohammadpour and Safaei (2022) developed a novel sulfonated carbon-coated magnetic catalyst (Fe_3_O_4_@C@OSO_3_H), which was used for the Pechmann condensation of phenol derivatives and β-ketoesters. The resulting yield values were as high as 98%, and the catalyst could be reused fifteen times with no significant loss in activity. Table 1 shows a summary of supported magnetic catalysts utilized for the synthesis of biodiesel.

**TABLE 1:** Recent findings on green biodiesel production using supported magnetic catalysts.

Entry	Feedstock/oil	Catalyst	Conditions (time, temperature, catalyst amount, and molar ratio of acid or oil to alcohol	Yield (*Y*/%) or conversion(*C*/%)	Times catalyst reused; yield	*E*a (KJ/mol)	Reference
1	Jatropha oil + methanol	CaSO_4_/Fe_2_O_3_–SiO_2_	4 h, 120°C, 12%, 1:9	Y = 94%	9 cycles; Y = 83%	\	Teo et al. (2019)
2	Rapeseed oil + methanol	Fe_3_O_4_-CeO_2_-25K	2 h, 65°C, 4.5%, 1:7	Y = 96.13%	5 cycles; Y = 80.94%	\	Ambat et al. (2019)
3	*Amygdalus scoparia* oil + methanol	NaOH/clinoptilolite–Fe_3_O_4_	2.5 h, 65°C, 0.5%, 1:10.43	Y = 91%	4 cycles; Y = 82%	9.21	Joorasty et al. (2021)
4	Fat + methanol	Fe_3_O_4_/Cs_2_O	5 h, 65°C, 7%, 1:21	Y = 97.1%	9 cycles; Y = 78%	43.8	Booramurthy et al. (2020)
5	*Pongamia pinnata* raw oil + methanol	CES-Fe_3_O_4_	2 h, 65°C, 2%, 1:12	Y = 98%	7 cycles; Y = 98%	\	Chingakham et al. (2023)
6	*Chlorella vulgaris* oil + ethanol	KF/KOH-Fe_3_O_4_	6 h, 25°C, 1.5%, 1:6	Y = 80%	Not reported	\	Farrokheh et al. (2021)
7	Used cooking oil + methanol	CaO-ZSM-5/Fe_3_O_4_	4 h, 65°C, 3%, 1:5	C = 83%	4 cycles; Y = 85%	\	Lani and Nagi, (2022)
8	Soybean oil + methanol	Co/Fe_2_O_3_-CaO	2.5 h, 70°C, 3%, 1:16	Y = 98.2%	5 cycles; Y = 78.8%	\	Xia et al. (2022)
9	Waste cooking oil + methanol	KOH/Fe_3_O_4_@MCM-41	3 h, 65°C, 8%, 1:40	Y = 93.95%	3 cycles; C>80%	115.79	Khakestarian et al. (2022)
10	Soybean oil + methanol	Na_2_CO_3_⋅H_2_O@BFD	2 h, 65°C, 7%, 1:15	Y = 100.0%	12 cycles; Y = 92.56%	\	Wang et al. (2022b)
11	Sunflower oil + methanol	Fe_2_O_3_/MS	4 h, 70°C, 0.03 g, 1:10(volume)	Y = 84.5%	Not reported	\	Nizam et al. (2022)
12	Glyceryl trioleate + methanol	Sulfamic acid-functionalized Fe/Fe_3_O_4_	20 h, 100°C, —, —	C = 100%	5 cycles; C = 95%	\	Wang et al. (2015)
13	Adipic acid + *n*-butanol	Sulfonated magnetic SiO_2_	4 h, 105°C, 2.95%, 1:3	C = 99%	6 cycles; C = 85.61%	\	Ke et al. (2019)
14	Acetic acid + methanol	Fe_2_O_3_–MCM-48–SO_4_	4.5 h, 60°C, 15 g/L, 1:10	C = 90%	5 cycles; C = 44.4%	29.077	Sharma et al. (2019)
15	Waste cooking oil + methanol	CSPA@Fe_3_O_4_	3 h, 65°C, 6%, 1:6	Y = 98%	9 cycles; Y = 91%	34.41	Changmai et al. (2021b)
16	Oleic acid + methanol	EFB-MCC/γ-Fe_2_O_3_	2 h, 60°C, 9%, 1:12	Y = 92.1%	5 cycles; Y = 77.6%	\	Krishnan et al. (2022)
17	Yeast oil + methanol	Fe_3_O_4_@SiO_2_-CHO	10 h, 55°C, 2.5 g, —	Y = 98.12%	10 cycles; Y = 90%	\	Cao et al. (2021)
18	Cottonseed oil + methanol	α-Fe_2_O_3_/AlOOH(γ-Al_2_O_3_)	3 h, 60°C, 3%, 1:6	Y = 100%	3 cycles; Y = 95%	57.4	Mohamed et al. (2020)
19	Waste cooking oil + methanol	α-Fe_2_O_3_/AlOOH	3 h, 60°C, 3%, 1:6	Y = 95%	4 cycles; Y = 91.3%	51.54	Mohamed and El-Faramawy, (2021)
20	Soybean oil + methanol	Fe_3_O_4_-poly(GMA-co-MAA)@ lipase	60 h, 40°C, 20%, 1:4	Y = 92.8%	5 cycles; Y = 79.4%	\	Xie and Huang, (2020)
21	Soybean oil + methanol	Fe_3_O_4_-poly(AGE-DVB-GMA)	8 h, 65°C, 7%, 1:20	Y = 92.6%	4 cycles; no significant decrease	\	Xie et al. (2021a)
22	Jatropha oil + methanol	Fe_3_O_4_@SiO_2_–SO_3_H	3.5 h, 80°C, 8%, 1:9	C = 98%	9 cycles; C = 81%	37.0	Changmai, et al. (2021a)
23	Oleic acid + methanol	SC-F-Plg-3	4 h, 65°C, 0.02 g, 1:55	C = 88.69%	5 cycles; C = 70.31%	\	Wu et al. (2022)
24	Cooking oil + methanol	Fe_3_O_4_@SiO_2_-APTES-L^AE^-Mo^VI^O_2_	0.75 h, RT, 0.04 g, 1:3	C = 99%	12 cycles; C = 92%	\	Mohammadpour and Safaei, (2022)

### 2.3 Magnetic catalysts functionalized with ionic liquids (ILs)

Recently, due to their highly tunable nature, low volatility, and strong chemical and thermal stability, ionic liquids (ILs) have been widely reported for use in the catalysis field (Sharma et al., 2022). Among these, many IL-functionalized magnetic catalysts have been tested for the production of biodiesel because of their unique properties and commercial availability. Fauzi et al. (2014) used oleic acid as raw material and 1-butyl-3-methylimidazolium tetrachloroferrate ([BMIM][FeCl_4_]) as a magnetic catalyst to prepare biodiesel by esterification, with a yield of methyl oleate of 83.4% under optimum conditions. In addition, the [BMIM][FeCl_4_] catalyst was reused for six runs with little loss; the activation energy of the esterification system was 17.97 kJ/mol.

A novel IL-functionalized magnetic catalyst was fabricated by covalent bonding of [SO_3_H-PIM-TMSP]HSO_4_ ILs onto mesoporous silica-modified Fe_3_O_4_ nanoparticles (FSS–IL) (Wu et al., 2014; Wan et al., 2015). The characterization results revealed that the FSS–IL catalyst possessed a uniform core–shell structure and high specific surface area. In the process of preparing biodiesel, the conversion was 93.5% after 4 h using oleic acid as a raw material. More importantly, this FSS–IL catalytic system remained active for six cycles. In another study, magnetically hydrophobic acidic polymeric ionic liquids (FnmS-PILs) were prepared and exhibited good activity and reusability (Zhang et al., 2018). Xie and Wang. (2020a) prepared a magnetic Fe_3_O_4_/SiO_2_-supported polymeric sulfonated ionic liquid (Fe_3_O_4_/SiO_2_-PIL) for simultaneous transesterification and esterification of low-cost oils, and the highest conversion obtained under optimal conditions was 93.3%. Additionally, the reusability study showed that the Fe_3_O_4_/SiO_2_-PIL could be recycled and reused five times. The higher activity and excellent reusability were attributed to the polymeric acidic ILs and porous magnetic nanoparticles. An immobilized dual acidic-ionic liquid on core–shell-structured magnetic silica was also prepared, and the as-prepared magnetic acid catalyst exhibited a large surface acidity of 3.93 meq H^+^/g, a strong magnetism of 27.5 emu/g, and achieved the highest conversion of biodiesel at 94.2%. The catalyst was reused for five runs, and the conversion still reached 86% (Xie et al., 2021).

Similar catalysts [NiFe_2_O_4_@BMSI]Br, Fe_3_O_4_@GO@PBIL, Fe_3_O_4_@SiO_2_@[C4mim]HSO_4_, Fe_3_O_4_@SiO_2_@PIL, and [BSO_3_HMIm][HSO_4_]@IRMOF-3 were also studied (Ding et al., 2021; Naushad et al., 2021; Yu et al., 2021; Zhao et al., 2021; Cheng et al., 2022). Among them, the magnetic [NiFe_2_O_4_@BMSI]Br catalyst was synthesized by an ion-exchange process, and the resulting catalyst had a BET surface area of 89.21 m^2^/g. Moreover, the [NiFe_2_O_4_@BMSI]Br catalyst attained a maximum yield of 86.4% for the transesterification of palm oil, and the catalytic activity was retained up to six cycles without obvious loss of yield (Naushad et al., 2021). Based on recent literature projections, ILs are expected to develop as potential acid materials for the synthesis of functionalized composite magnetic catalysts in the future.

### 2.4 Magnetic catalysts based on heteropolyacids

Heteropolyacids are inorganic compounds with a Keggin structure that acts as a strong Brønsted acid. Heteropolyacids have a low surface area and easily dissolve in polar solvents, so researchers bonded them to magnetic supports to overcome these problems. Wu et al. (2016a) investigated the application of magnetic material grafted onto a poly(phosphotungstate)-based acidic ionic liquid as a heterogeneous catalyst for the esterification of oleic acid. Under optimal conditions, the conversion of oleic acid reached 93.4%. More specifically, the catalyst exhibited good reusability after six runs using an external magnetic field.

As reported by Helmi et al. (2021), phosphomolybdic acid was supported on clinoptilolite–Fe_3_O_4_, and the prepared catalyst showed excellent activity (80% yield in 8 h at 75°C) and reusability in the production of biodiesel from *Salvia mirzayanii* oil. The HPA/clinoptilolite–Fe_3_O_4_ catalyst was able to recycle up to four times with minimal loss in activity. A magnetic heteropolyanion-based ionic liquid (MNP@HPAIL) was synthesized by Dadhania et al. (2021), and was evaluated for the esterification of oleic acid under ultrasonic irradiation. The maximum oleic acid conversion of 58% was reached, and the catalyst could be reused for six consecutive cycles.

On the same note, Zhang et al. (2021) immobilized a 12-tungstophosphoric acid (HPW)-based magnetic catalyst (Fe_3_O_4_@SBA-15@HPW and Fe_3_O_4_@SBA-15-NH_2_-HPW) for the production of biodiesel from palm oil with methanol. The synthesized magnetic catalysts have a high content of Brønsted acid sites due to the induction of HPW. In particular, the Fe_3_O_4_@SBA-15-NH_2_-HPW exhibited a high biodiesel yield of 91% under optimal reaction conditions, and also exhibited high reusability. Ghasemzadeh et al. (2022) adapted a cotton/Fe_3_O_4_@SiO_2_@H_3_PW_12_O_40_ magnetic nanocomposite to catalyze the transesterification of sunflower oil. The catalyst had an excellent magnetism of 45 emu/g and demonstrated a high FAME yield of 95.3% under optimum conditions. After four cycles of transesterification, the FAME yield was still relatively high at 85.5%. In addition, the physical properties of the synthetic biodiesel meet the ASTM and EU standards. According to the reported literature, heteropolyacids grafted onto magnetic supports can be an effective solution to overcome the loss of heteropolyacids.

### 2.5 MOF-based magnetic catalysts

Recently, metal–organic frameworks (MOFs), as a newly emergent type of stable and tunable material, have become promising magnetic catalysts and supports, and MOF derivatives have been used for heterogeneous catalysis. Wu et al. (2016b) investigated the ability of the Fe_3_O_4_@NH_2_-MIL-88B(Fe) catalyst to perform the esterification of oleic acid with ethanol. The Fe_3_O_4_@NH_2_-MIL-88B(Fe) catalyst had an acidity of 1.76 mmol/g and achieved a high yield of 93.2% at 90°C. Moreover, the Fe_3_O_4_@NH_2_-MIL-88B(Fe) catalyst could be recycled six times without significant loss of activity.

Xie’s group (Xie and Wan, 2018; Xie and Huang, 2019; Xie and Wang, 2020; Xie et al., 2021b) has studied biodiesel production from soybean oil and low-quality oils using magnetic Fe_3_O_4_@HKUST-1-ABILs, Fe_3_O_4_@MIL-100(Fe)/*Candida rugosa* lipase, CoFe_2_O_4_/MIL-88B(Fe)-NH_2_/(Py-Ps)PMo, and H_6_PV_3_MoW_8_O_40_/Fe_3_O_4_/ZIF-8 catalysts. Their results revealed that all magnetic catalysts exhibited good catalytic performance and excellent reusability. Thus, these MOF-based magnetic catalysts comprise an excellent potential alternative for processing low-quality oils into biofuels. In another study by Zhou’s group (Zhou et al., 2019; Zhou et al., 2023), a MIL-100(Fe) was embedded in magnetic Fe_3_O_4_ nanoparticles (Fe_3_O_4_/MIL-100(Fe), and the Fe_3_O_4_/MIL-100(Fe) composite exhibited unexpectedly high catalytic activity with a rosin conversion of 94.8% at 240°C. Furthermore, the Fe_3_O_4_/MIL-100(Fe) composite showed good stability and recyclability over six cycles. An annealed Fe_3_O_4_/MOF-5 was also synthesized and used to catalyze rosin esterification with glycerol. The highest conversion of 94.1% was attained in 2.5 h at 240°C, and the annealed catalyst showed excellent reusability.

A novel TiO_2_-decorated magnetic ZIF-8 nanocomposite (Fe_3_O_4_@ZIF-8/TiO_2_) was synthesized by Sabzevar et al. (2021). The as-prepared nanocomposite demonstrated excellent performance in the esterification of oleic acid (92.25% yield), which was mainly attributed to its acidic properties and large surface area. After five cycles, the yield of biodiesel was still 77.22%. Abdelmigeed et al. (2021a), Abdelmigeed et al. (2021b) prepared NaOH/magnetized ZIF-8 catalysts for the production of high-quality biodiesel from a blend of sunflower and soybean oil with ethanol. The transesterification reaction with the blended oil produced 70% biodiesel in 1.5 h at 75°C. The ethanolysis reaction followed a pseudo-second-order kinetic model, and the activation energy was calculated as 77.27 kJ/mol.

In another important area of catalyst research, MOFs were pyrolized at various temperatures to act as self-sacrificial templates for the synthesis of structured nanoporous metal oxides (Reddy et al., 2020). Li et al. (2019), Li et al. (2020), Li et al. (2021) reported on a series of magnetic catalysts based on MOF derivatives (MM–SrO, magnetic CaO-based catalyst, carbonized MIL-100(Fe) supporting ammonium sulfate), and the physical, chemical, and thermal properties of the MOF-derived magnetic catalysts were evaluated. The researchers discovered that these catalysts exhibited strong magnetism and excellent catalytic activity and could be easily separated by an external magnetic field after each cycle. In another study, a bifunctional magnetic catalyst with various coordination states of Co and non-coordinated N sites was developed by Guo et al. (2022). The prepared bifunctional magnetic catalyst (550–30) was evaluated for biodiesel production from microalgal lipids. It had a high conversion efficiency of 96.0%, owing to the generated structural defects that formed a mesopore-dominated structure in the bifunctional magnetic catalyst. Also, the catalyst could be magnetically separated and reused for six cycles with a conversion efficiency of 89.7%.

## 3 Summary and outlook

In the field of catalysis, magnetic catalysts promote catalytic reactions efficiently and their strong magnetic properties allow them to be easily reused, which make magnetic catalysts more cost-effective and efficient when used in industrial catalysis. The current mini-review highlights recent applications of magnetic catalysts and their functionalized magnetic materials utilized for biodiesel production. Although remarkable progress has been achieved in the area of magnetic catalyst research, there are still some limitations that need to be overcome by continuing design improvements. The catalytic mechanisms and deactivation processes are not well understood, supported magnetic catalysts show weak interactions between active ingredients and magnetic supports, and the complex synthesis processes for some magnetic catalysts need to be simplified. Thus, future investigation into the preparation methods, performance, mechanisms, and economics of the magnetic catalyst is essential to correct the present issues. In light of the current evidence, however, we strongly believe that the integrated development of novel magnetic catalysts will play a key role in further developing a cost-effective biorefinery industry.
